# Calcium phosphate injection of symptomatic bone marrow lesions of the knee: what is the current clinical evidence?

**DOI:** 10.1186/s43019-019-0013-3

**Published:** 2020-01-01

**Authors:** D. S. Angadi, D. Edwards, J. T. K. Melton

**Affiliations:** 1 Department of Orthopaedic Surgery, Cheltenham General Hospital, Sandford Road, Cheltenham, Gloucestershire GL53 7AN UK; 2 Department of Trauma and Orthopaedics, Addenbrookes Hospital, Cambridge, UK

**Keywords:** Knee, Bone marrow, Lesion, Calcium phosphate, Subchondroplasty, Osteoarthritis

## Abstract

**Background:**

Chronic bone marrow lesions (BML) in the weight-bearing portions of the knee are often associated with symptomatic degenerative arthritis resulting in pain and dysfunction. Injection of bone substitute material like calcium phosphate has been described. Whilst some studies have reported encouraging results others have shown limited benefit of this technique.

**Aim:**

The aim was to collate the available evidence on the injection of calcium phosphate and systematically evaluate the results to answer the questions encountered in clinical decision making: (1) does it provide effective long-lasting pain relief to avoid further surgical intervention? (2) which factors (patient/surgical) significantly influence the outcome? and (3) does it adversely affect the outcomes of subsequent arthroplasty?

**Methods:**

A literature search was performed to identify the studies describing the clinical outcomes of calcium phosphate injection for treatment of BML. We evaluated the reported clinical outcomes with respect to pain, function and complications. Isolated case reports and studies with no objective assessment of clinical outcomes were excluded.

**Results:**

We noted 46 articles in the current literature of which 8 described clinical outcomes of calcium phosphate injection. Mean (plus/minus SD) score on the visual analog scale (VAS) has been reported to improve from 7.90 (± 0.38) to 2.76 (± 0.90), whereas the International Knee Documentation Committee (IKDC) score improved from 30.5 (SD not reported (NR)) to 53.0 (SD NR). Pre and post procedure Short form survey (SF-12) scores were 29.8 (SD NR) and 36.7 (SD NR), respectively. In one study, scores on the Tegner Lysholm knee scoring scale improved in 12 out of 22 patients, whereas the remainder had no change in symptoms. Extravasation of calcium phosphate into the joint was the most common complication, whereas no adverse effect has been reported on subsequent arthroplasty.

**Conclusion:**

Limited data from the published studies would suggest that calcium phosphate injection of BML may potentially improve pain and function. However, no evidence is currently available to clearly identify patient/surgical factors that may influence the long-term outcomes of this procedure. Hence pragmatic, prospective studies with stratified patient cohorts and robust reporting of outcome measures are essential to improve the understanding of the indications and clinical effectiveness of this novel procedure.

## Introduction

Chronic bone marrow lesions (BML) in the weight-bearing portions of the knee are often associated with symptomatic degenerative arthritis resulting in pain and dysfunction [1–4]. BML may not be apparent on radiographs and are diagnosed on magnetic resonance imaging (MRI) using fat-saturated T2-weighted, proton density-weighted or short tau inversion recovery (STIR) image sequences [5, 6]. They appear as poorly defined areas of high signal intensity in the subchondral region [5–8]. These lesions represent sites of increased mechanical stress [9] with subchondral hyperintense signal suggestive of an insufficiency fracture [5, 6]. They may also be correlated with common degenerative conditions of the knee such as meniscal tears [2], cartilage deterioration [5, 6, 9], subchondral bone attrition and cyst formation [3], mechanical malalignment [4] and ultimately, progression of osteoarthritis of the knee [2].

Management of symptomatic BML in patients with degenerate change of the knee using percutaneous injection of bone substitute material like calcium phosphate has been described in the literature [10–12]. The proprietary term Subchondroplasty® (SCP) (Zimmer Knee Creations; West Chester, PA, USA) refers to a novel technique in which a BML is injected with a bone substitute material composed of calcium phosphate, using fluoroscopic guidance [13]. The perceived advantages of Subchondroplasty® in managing these patients include the minimally invasive percutaneous technique [14], the potential for improved structural integrity and biomechanical strength of the subchondral bone from the injected calcium phosphate, which may additionally function as a porous osteoconductive scaffold allowing formation of new bone capable of sustaining load [1, 12, 13]. Since the description of the initial procedure in 2008 for chronic BML around the knee, the indications for this technique has been extended by some authors to include osteochondritis dessicans of the lateral tibial plateau in skeletally immature patients [15], subchondral bone marrow oedema of the talar dome due to ankle instability [16], osteochondral lesion of the talus [16] and BML of the shoulder and hip [2]. Initial studies of this procedure as reported in the literature have had encouraging results [1, 11, 13]. However, it is noted that these studies involve small cohorts of patients with limited follow up. Furthermore, some authors have reported unsatisfactory clinical results questioning the efficacy of this procedure in advanced osteoarthritis of the knee [17], femoral BML and lesions with mild bone marrow oedema [18]. It has been reported that there is considerable variation in the biomechanical properties of the commercially available calcium phosphate used as injectable bone substitute material during this procedure [19].

The primary objective of this review was to collate the available clinical studies in the published literature and review the results of percutaneous injection of bone substitute material like calcium phosphate including Subchondroplasty®. The secondary objective was to answer the following questions encountered in the clinical decision-making process of managing patients with BML associated with osteoarthritis of the knee:

1. Does calcium phosphate injection or Subchondroplasty® provide effective long-lasting pain relief to avoid further surgical intervention?
2. Which factors (patient/surgical) significantly influence the outcome following this type of procedure?
3. Does calcium phosphate injection or Subchondroplasty® adversely affect the outcomes of subsequent arthroplasty?

## Materials and methods

### Literature search and databases

A literature search of all the available evidence was undertaken (June 2019) using the healthcare database website (https://hdas.nice.org.uk). The databases searched were Medline, CINAHL, Embase and the Cochrane library.

Search criteria included boolean statements and the wildcard symbol (femur* OR femoral* OR tibia* OR tibial* OR knee*) AND (bone* OR marrow*) AND (lesion* OR edema* OR oedema*) AND (subchondroplasty*)”. The Cochrane database was reviewed for relevant articles.

An adjunctive bibliography search was undertaken to identify additional relevant articles. Subsequently duplicate articles were excluded and the clinical results of calcium phosphate injection including Subchondroplasty® were reviewed. Animal model and in vitro studies were excluded.

## Results

**Fig. 1:**
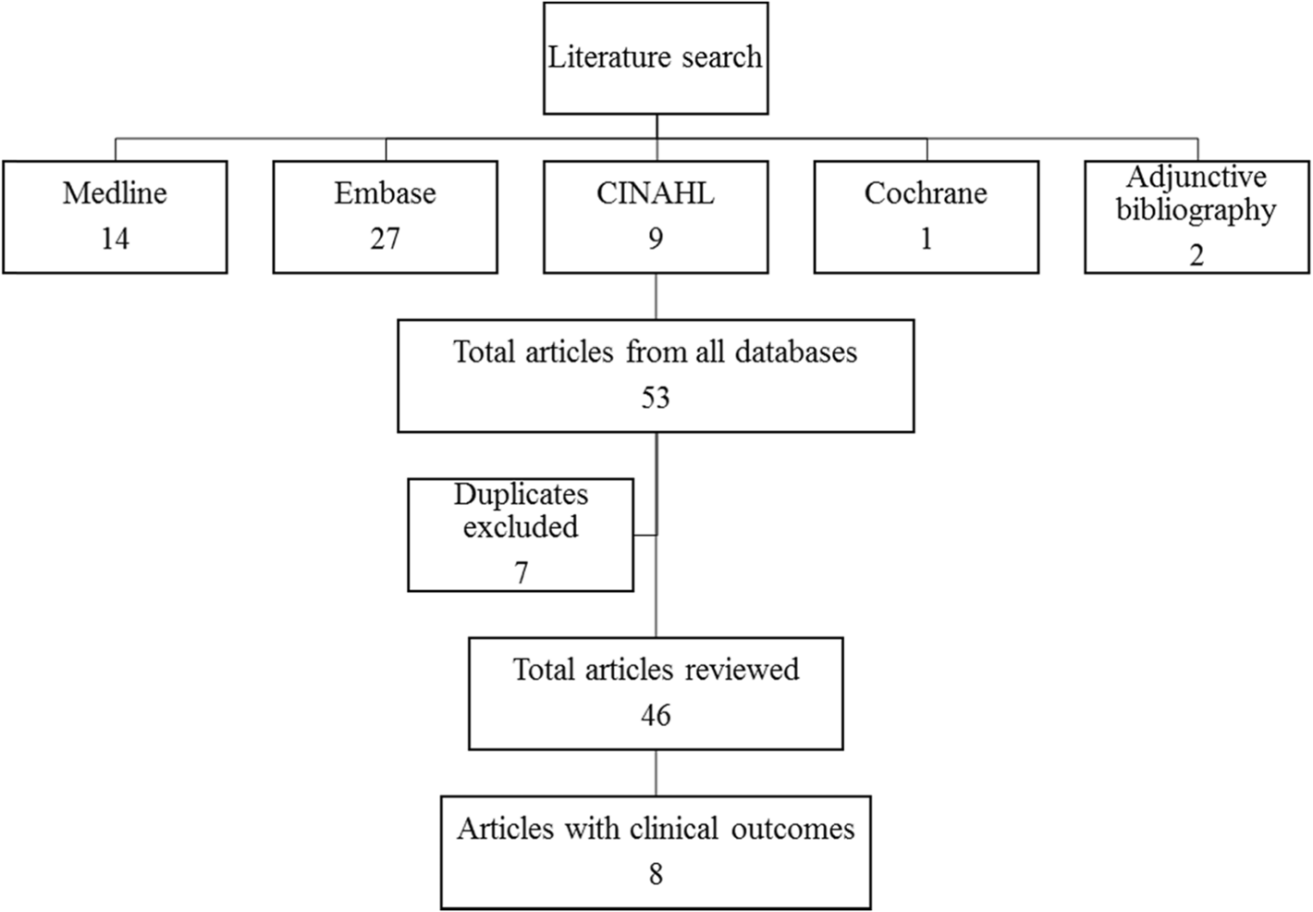
Results of literature search

The aforementioned database search and adjunctive bibliography search returned 53 relevant articles of which 7 articles were noted to be duplicates (Fig. 1). Thus, a total of 46 relevant articles were identified in the current literature: amongst these, 8 articles described the clinical results of calcium phosphate injection/subchondroplasty and were selected for further review. Details of the eight articles that describe the clinical outcomes of subchondroplasty are provided in Tables 1 and 2. The salient points are discussed below.

**Table 1:** Details of clinical studies

Author and year	Number of patients	Age at presentation/ mean (±SD)/median (range)	Duration of nonoperative treatment (pre-procedure)	Mechanical axis evaluated preoperatively (Y/N)	Bone substitute material used	Postoperative regimen Weight- bearing	Duration of follow up (months) Mean (±SD)/median (range)	Outcome measures	Complications (number)
Conway et al. [20] 2019	1	54	NR	N	AccuFill®	Toe-touch	24	VAS	Increased pain
Bonadio et al. [12] 2017	5	67.7(±9.67)	3	Y	Graftys HBS®	Full	6 (±NR)	KOOS VAS	Graft extravasation (1)
Byrd et al. [21] 2017	133	57 (38–84)	NR	N	AccuFill®	NR	32.1 (24–43)	VAS	NR
Cohen et al. [11] 2016	66	55.9 (35–76)	2	Y	AccuFill®	As tolerated + crutches	24 (NR)	VAS IKDC	Persistent drainage (1) Deep vein thrombosis (1)
Chatterjee et al. [17] 2015	22	53.5 (38–70)	3	Y	AccuFill®	Partial (2/52)	12 (6–24)	KOOS T-L	NR
Nevalainen et al. [7] 2015	2	42 58	8 NR	N N	AccuFill® AccuFill®	NR NR	6 14	NR NR	NR NR
Farr et al. [13] 2013	59	55.6 (35–76)	6	Y	AccuFill®	As tolerated + crutches	14.7 (±NR)	VAS IKDC SF12	Extravasation (N/R) Significant pain treated with nerve block (NR)
Davis et al. [22] 2015	50	55 (36–82)	NR	NR	NR	NR	14.6 (12.9–25.1)	VAS	NR

*SD* standard deviation, *NR* not reported, *Y* yes, *N* no, *VAS* visual analog scale, *KOOS* Knee injury and Osteoarthritis Outcome Score, *IKDC* International Knee Documentation Committee, *T-L* Tegner Lysholm Knee Scoring Scale, *SF-12* Short Form Survey Score

**Table 2:** Reported clinical outcomes of calcium phosphate injection including Subchondroplasty®

Author and year	Number of patients	Age at presentation /mean (±SD)/ median (range)	Duration of follow up (months) Mean (±SD)/ median (range)	Outcome measure	Pre-procedure		Post-procedure	
					Time point(s) (weeks/months)	Score Mean (±SD)	Time point(s) (weeks/months)	Score Mean (±SD)
Bonadio et al. [12] 2017	5	67.7(±9.67)	6 (±NR)	VAS	1 week	7.8 (±NR)	1 week 3 weeks 6 weeks 12 weeks 24 weeks	2.8 (±NR) 3.0 (±NR) 2.8 (±NR) 1.8 (±NR) 0.6 (±NR)
Byrd et al. [21] 2017	133	57 (38–84)	32.1 (24–43)	VAS	NR	8.3 (±NR)	NR	3.4 (±NR)
Cohen et al. [11] 2016	66^a^	55.9 (35–76)	24 (NR)	VAS	NR	7.6 (±NR)	6 months 24 months	3.3 (±NR) 3.2 (±NR)
Davis et al. [22] 2015	50	55 (36–82)	14.6 (12.9–25.1)	VAS	NR	8.3 (±NR)	NR	3.6 (±NR)
Farr et al. [13] 2013	59	55.6 (35–76)	14.7 (±NR)	VAS	NR	7.5 (±NR)	6 months	3.1 (±NR)

*SD* standard deviation, *NR* not reported, *NA* not applicable, *VAS* visual analog scale, *KOOS* Knee injury and Osteoarthritis Outcome Score, *IKDC* International Knee Documentation Committee, *T-L* Tegner Lysholm Knee Scoring score, *SF-12* Short Form Survey score)

^a^ Limited patients at follow up: 6 months, *n* = 6, 12 months, *n* = 5, 24 months, *n* = 1

### Patient demographics and physiology

Wide variation was noted in the patient population with the youngest patient reported to be 35 years of age at the time of the procedure [11, 13] whereas the oldest patient was 82 years [22].

Body mass index (BMI) is also reported inconsistently in the literature. Some studies [1, 7, 11, 13, 17] provide the details of BMI for their patient cohort whereas other studies [12, 18, 21, 22] do not provide any information on this vital parameter.

### Indications and severity of osteoarthritis

There is good agreement in the literature for treating chronic painful bone marrow lesions noted in knee arthritis with injection of calcium phosphate [1, 7, 10, 12, 13, 22]. However, there is a lack of consensus on the degree of radiographic severity of knee arthritis for which this procedure is effective. Whilst some authors [11] have included patients with Kellgren-Lawrence (K-L) grade 4 arthritis, other authors consider it a contraindication [12, 17].

### Mechanical parameters (range of motion/lower limb alignment)

Inconsistency is noted in the reported literature on mechanical parameters such as range of motion and mechanical axis in patients undergoing subchondroplasty. Several studies evaluated mechanical axis preoperatively and excluded patients with > 8° of varus/valgus mal-alignment [1, 11, 12, 17]; however, some authors have not stated the mechanical parameters in their series [1, 13, 18].

### Surgical technique and postoperative regimen

There is considerable variation in the reported surgical technique used to perform the injection of bone substitute material like calcium phosphate to treat BML associated with knee arthritis. These includes (1) use of different types of commercially available injectable calcium phosphate such as Graftys HBS® (Graftys, Aix en Provence, France) [12], AccuFill® (Zimmer Knee Creations; West Chester, PA, USA) [7, 11, 13, 17]; (2) injection of calcium phosphate into BML under fluoroscopic guidance alone [7, 12] or combining it with arthroscopic assessment of chondral surfaces; (3) adjunctive arthroscopic procedures [1, 10, 11, 13, 17] and viscosupplementation [17, 22] and (4) injection of calcium phosphate with [10, 17] and without [12, 13] the use of a targeting jig/device.

Additionally, variable perioperative and postoperative regimens have been described for this procedure. These include (1) weight-bearing regimens ranging from full weight-bearing [12], weight-bearing as tolerated with crutches for 1 week [11] or for 2 weeks postoperatively [10, 13] to partial weight-bearing for 2 weeks postoperatively [17] and (2) type of analgesia used is variable ranging from none [17], oral/parenteral dipyrone with tramadol [12], acetaminophen with anti-inflammatory medications viz. indomethacin/ibuprofen [17], opiates such as. oxycodone/hydrocodone [17] and oral opiates alone [10, 13] to not reported [1, 11].

### Outcome measures and follow-up duration

Different outcome measures have been used to describe the results of this procedure (Tables 1, 2 and 3). These include visual analog scale (VAS) score for pain [1, 11, 12, 22], Knee injury and Osteoarthritis Outcome Score (KOOS) [12, 17], Lysholm and Tegner score [17], International Knee Documentation Committee (IKDC) score [11, 13] and the Short Form Survey (SF-12) score [13]. The duration of follow up ranged from 6 months [12] to a maximum of 43 months [21].

**Table 3:** Reported clinical outcomes of calcium phosphate injection including Subchondroplasty®

Author and year	Number of patients	Age at presentation /mean (±SD)/ median (range)	Duration of follow up (months) Mean (±SD)/ median (range)	Outcome measure	Pre-procedure		Post-procedure	
					Time point(s) (weeks/months)	Score Mean (±SD)	Time point(s) (weeks/months)	Score Mean (±SD)
Chatterjee et al. [17] 2015	22^a^ 5/22 1/22	53.5 (38–70)	12 (6–24)	KOOS	NR NR NR	39.28 (±26.52) 42.56 (±18.62) 61.30 (±NA)	6 months^a^ 12 months^a^ 24 months^a^	79.68 (±22.69) 86.78 (±10.71) 62.50 (±NA)
Cohen et al. [11] 2016	66^a^ 35/66 26/66	55.9 (35–76)	24 (NR)	IKDC	NR	30.5 (±NR)	6 months 24 months	47.7 (±NR) 48.3 (±NR)
Farr et al [13] 2013	59	55.6 (35–76)	14.7 (±NR)	IKDC	NR	30.6 (±NR)	6 months	53.0 (±NR)
Chatterjee et al. [17] 2015	22^a^ 5/22 1/22	53.5 (38–70)	12 (6–24)	T-L	NR NR NR	52.00 (±15.47) 49.20 (±6.53) 44.00 (±NA)	6 months^a^ 12 months^a^ 24 months^a^	86.50 (±12.72) 90.20 (±7.16) 47.00 (±NA)
Farr et al. [13] 2013	59	55.6 (35–76)	14.7 (±NR)	SF12	NR	29.8 (±NR)	6	36.7 (±NR)

*SD* standard deviation, *NR* not reported, *NA* not applicable, *VAS* visual analog scale, *KOOS* Knee injury and Osteoarthritis Outcome Score, *IKDC* International Knee Documentation Committee, *T-L* Tegner Lysholm Knee Scoring score, *SF-12* Short Form Survey score)

^a^ Limited patients at follow up: 6 months, *n* = 6, 12 months, *n* = 5, 24 months, *n* = 1

### Complications

Extravasation of calcium phosphate into soft tissues or the joint has been reported [10, 12, 13]. Other reported complications include significant postoperative pain [10, 13, 20], which may last up to 12 months [20], persistent drainage at the injection site [11] and deep vein thrombosis [11].

## Discussion

### Bone marrow lesions

Bone marrow lesions associated with osteoarthritis of the knee have been well-described in the current literature [1–3, 6, 8, 9, 23]. It has been suggested that altered biomechanics due to abnormal joint loading and the corresponding subchondral BML contribute to the aetiopathogenesis of osteoarthritis [3, 23]. Chronic BML around the weight-bearing regions of the knee are often associated with symptomatic degenerative arthritis resulting in pain [1–4]. Management of these lesions has been broadly described using non-operative and operative methods alongside osteoarthritis of the knee. Non-operative methods including oral nonsteroidal anti-inflammatory medications have been demonstrated to provide symptomatic relief in patients with osteoarthritis and BML [24]. Use of other medications like bisphosphonates [25] and prostacyclins [26, 27] have been described in the management of BML; however, the long-term efficacy of these measures is limited [2]. Operative methods such as core decompression [28, 29] and high tibial osteotomy [30, 31] have been proposed as treatment options for BML associated with osteoarthritis of the knee.

### Surgical technique - paucity of literature

Despite only limited studies being available in the decade since this procedure was first described, the indications have been widely extended to include osteochondritis dessicans of the lateral tibial plateau in skeletally immature patients [15], subchondral bone marrow oedema of the talar dome due to ankle instability [16], osteochondral lesion of the talus [16] and BML of the shoulder and hip [2].

Currently there is a lack of consensus in the literature on the surgical technique for this procedure. Some authors [1, 10, 11, 13, 17] performed adjunctive arthroscopic procedures to address meniscal and chondral lesions in addition to the injection of calcium phosphate. The influence of these adjunctive procedures on the functional improvement in clinical outcomes is difficult to quantify given the small patient cohorts and limited follow up. Careful interpretation of the results from these studies is essential. Intraoperative localisation of BML has been aided using fluoroscopy and targeting devices [17]; however, the use of targeting devices is not consistently reported in the literature [12].

### Bone substitute material - nanocrystalline calcium phosphate

As highlighted above, different types of calcium phosphate have been described for injection of painful BML. It has been demonstrated that different types of commercially available calcium phosphate used as bone substitute material have considerable variation in their in vitro injectability and biomechanical properties [19]. Using synthetic (polyurethane foam sheets) and cadaveric cancellous bone blocks, Colon et al. [19] noted that only AccuFill® (Zimmer, Inc.) and StrucSure™ CP (Smith and Nephew) were able to flow into a closed structure such as cancellous bone. Furthermore, they observed that amongst the eight bone substitute materials tested, AccuFill® had the lowest injection force and achieved desired fill of the void. It is interesting to note that both AccuFill® and StrucSure™ CP are composed of nanocrystalline calcium phosphate [19]. Hence the type of calcium phosphate used to inject BML and the pressure used may have a critical influence on the outcome of Subchondroplasty®. The surgical experience of the surgeon performing Subchondroplasty® may be an additional factor.

### Imaging features after subchondroplasty

Subsequent to injection of BML with calcium phosphate imaging modalities like magnetic resonance imaging (MRI) have been used to evaluate the subchondral bone. The injected calcium phosphate has been described to demonstrate a low signal surrounded by hyperintense rim on T2-weighted images [5, 7].

### Lack of consensus on outcome measures

There is considerable variation in the literature on the outcome measures used to describe the clinical results of calcium phosphate injection into BML (Tables 2 and 3). Analysis of the different outcome measures is presented in Table 3. The VAS score for pain has been demonstrated to significantly decrease following Subchondroplasty® [1, 11, 12, 22]. Some authors have reported an improvement in the KOOS [12, 17] and IKDC score [11, 13], whereas the Tegner Lysholm score [17] was noted to deteriorate in a different study. This lack of consensus is a major limitation in the interpretation of results. Hence it is essential to establish a set of robust clinical and radiological outcome measures that will be consistently used in future studies to report the results of this procedure.

### Limited follow up and natural history of osteoarthritis

The maximum reported follow up after this procedure is 43 months [21]. Given the long-standing natural history of osteoarthritis, the limited follow up reported in the current literature is a further limitation in the interpretation of results.

### Morbidity and implications for future procedures after subchondroplasty

Overall, the injection of calcium phosphate into BML around the knee appears to be associated with limited morbidity. Extravasation of the material into the soft tissues or into the joint have been reported [10, 12, 13]. However, calcium phosphate is a hydrophilic material and it has been suggested that the residual material can be removed arthroscopically using standard instruments [13].

The perceived advantage of injectable calcium phosphate is that it may be able to function as a scaffold following the setting process involving an endothermic reaction [13, 14]. This enables bone growth providing structural support [11]. Nonetheless there has been concern that this alteration in bony architecture may limit future procedures like arthroplasty. This has been addressed in a recent study by Yoo et al. [32]. They reported no increase in surgical complexity among patients undergoing arthroplasty following Subchondroplasty®. Furthermore, they noted that these patients had a similar postoperative Oxford score to their control group.

### Lack of comparative studies for novel techniques

From a basic science perspective, calcium phosphate injection of BML has been suggested to play a mechanical role providing structural support [1, 11, 13]. BML are sites of altered bone remodelling activity in which the mesenchymal stem/stromal cells (MSCs) play a crucial role [33]. It has been demonstrated that as the frequency of symptomatic BML increases with progression of knee osteoarthritis, necessitating arthroplasty [34]. Hence novel techniques using biological agents such as platelet-rich plasma (PRP) or dual-action antiresorptive agents such as strontium ranelate delivered directly into BML has been proposed to improve bone homeostasis and joint preservation [34]. However, there is no study in the current literature comparing these novel techniques with calcium phosphate injection of BML.

## Conclusions

The limited data from the current studies would suggest that injection of calcium phosphate in BML including Subchondroplasty® may improve pain and function. The procedure is associated with limited morbidity and it does not appear to negatively influence the outcome of subsequent arthroplasty. However, the type of injectable calcium phosphate and adjunctive procedures undertaken may have an influence on the clinical outcome. Hence pragmatic, prospective studies with stratified patient cohorts, longer follow-up duration and robust outcome measures are essential to improve the current understanding of the indications and the clinical effectiveness of this procedure.

## Data Availability

Presented in the manuscript.

## Abbreviations

BMI: Body mass index
BML: Bone marrow lesion
IKDC: International Knee Documentation Committee
KOOS: Knee injury and Osteoarthritis Outcome Score
MRI: Magnetic resonance imaging
MSCs: Mesenchymal stem/stromal cells
NR: Not reported
PRP: Platelet-rich plasma
SCP: Subchondroplasty
SD: Standard deviation
SF-12: Short Form Survey Score
STIR: Short tau inversion recovery
T-L: Tegner Lysholm Knee Scoring Scale
VAS: Visual analog scale
